# Tar DNA Binding Protein-43 (TDP-43) Associates with Stress Granules: Analysis of Cultured Cells and Pathological Brain Tissue

**DOI:** 10.1371/journal.pone.0013250

**Published:** 2010-10-11

**Authors:** Liqun Liu-Yesucevitz, Aylin Bilgutay, Yong-Jie Zhang, Tara Vanderwyde, Allison Citro, Tapan Mehta, Nava Zaarur, Ann McKee, Robert Bowser, Michael Sherman, Leonard Petrucelli, Benjamin Wolozin

**Affiliations:** 1 Department of Pharmacology, Boston University School of Medicine, Boston, Massachusetts, United States of America; 2 Department of Neuroscience, Mayo Clinic, Jacksonville, Florida, United States of America; 3 Department of Neurology, Boston University School of Medicine, Boston, Massachusetts, United States of America; 4 Department of Pathology, University of Pittsburgh School of Medicine, Pittsburgh, Pennsylvania, United States of America; 5 Department of Biochemistry, Boston University School of Medicine, Boston, Massachusetts, United States of America; Mental Health Research Institute of Victoria, Australia

## Abstract

Tar DNA Binding Protein-43 (TDP-43) is a principle component of inclusions in many cases of frontotemporal lobar degeneration (FTLD-U) and amyotrophic lateral sclerosis (ALS). TDP-43 resides predominantly in the nucleus, but in affected areas of ALS and FTLD-U central nervous system, TDP-43 is aberrantly processed and forms cytoplasmic inclusions. The mechanisms governing TDP-43 inclusion formation are poorly understood. Increasing evidence indicates that TDP-43 regulates mRNA metabolism by interacting with mRNA binding proteins that are known to associate with RNA granules. Here we show that TDP-43 can be induced to form inclusions in cell culture and that most TDP-43 inclusions co-localize with SGs. SGs are cytoplasmic RNA granules that consist of mixed protein - RNA complexes. Under stressful conditions SGs are generated by the reversible aggregation of prion-like proteins, such as TIA-1, to regulate mRNA metabolism and protein translation. We also show that disease-linked mutations in TDP-43 increased TDP-43 inclusion formation in response to stressful stimuli. Biochemical studies demonstrated that the increased TDP-43 inclusion formation is associated with accumulation of TDP-43 detergent insoluble complexes. TDP-43 associates with SG by interacting with SG proteins, such as TIA-1, via direct protein-protein interactions, as well as RNA-dependent interactions. The signaling pathway that regulates SGs formation also modulates TDP-43 inclusion formation. We observed that inclusion formation mediated by WT or mutant TDP-43 can be suppressed by treatment with translational inhibitors that suppress or reverse SG formation. Finally, using Sudan black to quench endogenous autofluorescence, we also demonstrate that TDP-43 positive-inclusions in pathological CNS tissue co-localize with multiple protein markers of stress granules, including TIA-1 and eIF3. These data provide support for accumulating evidence that TDP-43 participates in the SG pathway.

## Introduction

TDP-43 is the principle protein component of inclusions in ALS and ubiquitin positive frontotemporal lobar degeneration (FTLD-U) [1]. Currently more than thirty mutations in TDP-43 have been identified in familial and sporadic ALS [2], [3]. Mutations in TDP-43 are considered as a major cause of familial ALS. TDP-43 is a 414 amino acid nuclear protein encoded by the TARDBP gene on chromosome 1. Although it is ubiquitously expressed in all tissues, it highly expresses in the brain and kidney [4]. TDP-43 is an mRNA binding protein that plays important functions in regulating mRNA metabolism involved in several functions, including transcriptional repression, exon skipping and RNA splicing [5], [6]. It contains two RNA binding domains and a glycine rich domain at the C terminus. The two RNA recognition motifs and the C terminal glycine-rich domains in the TAR-DNA appear to be important for its interactions with nucleic acids [5], [6]. TDP-43 predominantly expresses in the nucleus where it exerts its biological functions, but in pathological tissue TDP-43 is aberrantly processed and forms inclusions in the cytoplasm. The mislocalization of TDP-43 in the cytoplasm highlights important gaps in our knowledge of TDP-43 biology.

mRNA binding proteins facilitate mRNA trafficking from the nucleus to the cytoplasm as part of the biological machinery that regulates mRNA metabolism, such as RNA decay and protein translation. RNA decay is a constitutive process that occurs in cytoplasmic compartments termed processing bodies (P-bodies). However, under stressful conditions mRNA binding proteins consolidate mRNA in cytoplasmic compartments, termed the stress granules (SGs); this recruitment is mediated by multiple proteins, including T-cell intracellular antigen 1 (TIA-1), RasGAP-associated endoribnuclease (G3BP), elongation initiation factor 3 (eIF3) and poly-A binding protein (PABP) [7]. SGs function in part to triage RNA and sequester transcripts not needed for coping with the stress [7]. The mechanism of SG formation is striking because it results from the regulated, reversible aggregation process of mRNA binding proteins with prion-like domains, such as TIA-1, TIAR and G3BP[8].

TDP-43 inclusions isolated from brains of subjects with FTLD-U contain full-length TDP-43 as well as C-terminal cleavage fragments that are approximately 25 and 35 KD in size [1]. Recent studies with cell lines indicate that TDP-43 can form cytoplasmic inclusions when expression is forced to the cytoplasm by removal of the nuclear localization signal [9], [10]. TDP-43 inclusions also occur upon apoptosis, possibly because the caspase-generated cleavage fragments of TDP-43 have a strong tendency to aggregate [9], [11], [12], [13]. Increasing evidence suggests that TDP-43 cytoplasmic inclusions observed in cell culture are SGs [14], [15], but the mechanisms by which TDP-43 might associate with SG are unknown. In addition, also unknown are questions such as whether SG biology contributes to ALS and how disease-linked TDP-43 mutations might participate in this process. We now report that TDP-43 co-localizes with SGs in cells and in affected CNS tissue from patients with ALS or FTLD-U. We also report that TDP-43 binds to TIA-1, an intrinsic SG protein, which provides a potential mechanism for association of TDP-43 with SGs in ALS and FTLD-U. In additions, we report that disease-linked mutations in TDP-43 increase the formation of cytoplasmic TDP-43 inclusions that are co-localized with SG markers.

## Results

### Over-expressed TDP-43 forms cytoplasmic inclusions that co-localize with SG

**Figure 1:**
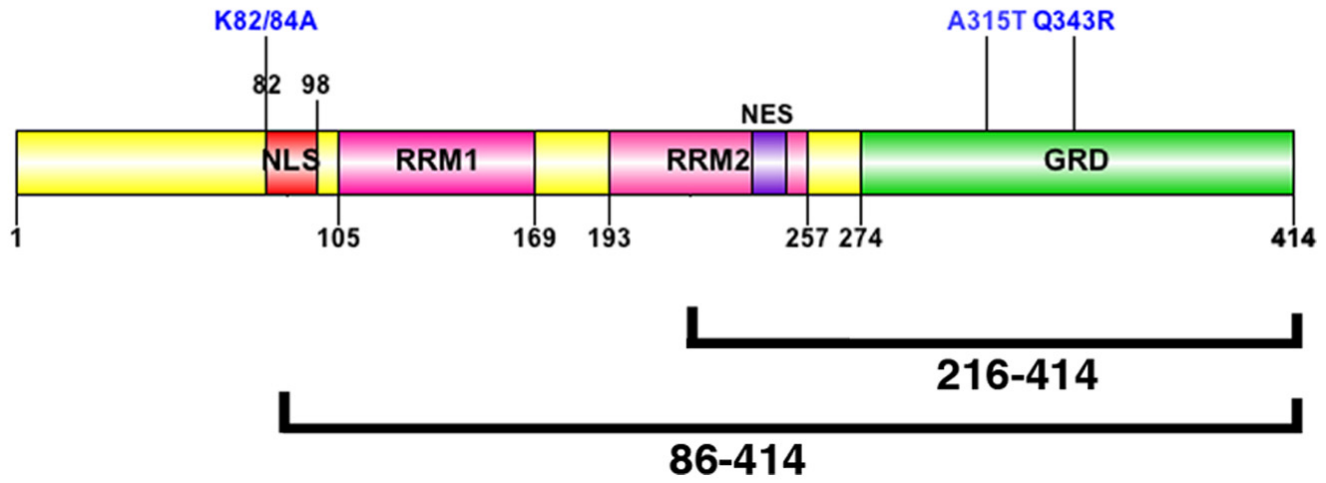
TDP-43 constructs: Diagram showing the TDP-43 constructs used in this report. EGFP, Enhanced Green Fluorescent Protein; RRM, RNA recognition motif; GRR, Glycine rich domain; NLS, nuclear localization signal; NES, nuclear export signal.

**Figure 2:**
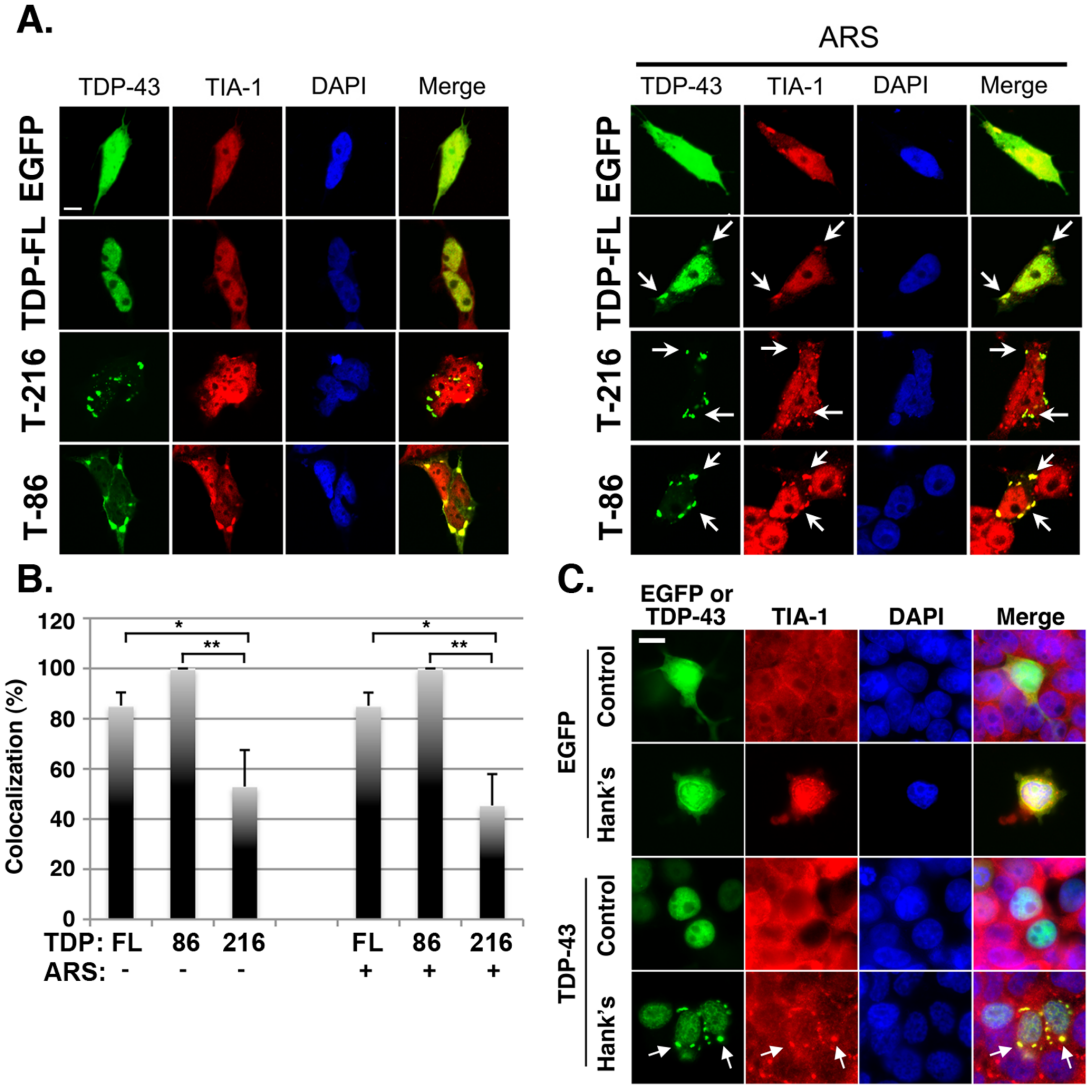
TDP-43 inclusions co-localize with SG proteins. A.) Cellular distribution of TDP-43::EGFP constructs in human neuroblastoma BE-M17 cells under basal conditions and after treatment with arsenite; the cells are co-labeled with anti-TIA-1 antibody (red, labeling SGs) and with DAPI (blue, labeling nuclei). Basal conditions (left panel): WT TDP-43 (T-FL) typically shows diffuse nuclear expression, while TDP-43_216–414_ (T-216) and TDP-43_86–414_ (T-86)::EGFP constructs form cytoplasmic and nuclear inclusions. TDP-43 reactivity co-localizes with RNA binding protein, TIA-1. Arsenite treatment (right panel): Treatment with arsenite (0.5 mM, 1 hr) induces some TDP-43::EGFP to translocate to the cytoplasm where it forms inclusions (arrows) that co-localize with TIA-1, indicating that the inclusions are SGs. The TDP-43_216–414_ and TDP-43_86–414_::EGFP constructs form inclusions in the cytoplasm (arrows) and nucleus, but only inclusions generated by the TDP-43_86–414_ construct are increased by arsenite. The inclusions present in arsenite treated cells co-localize with TIA-1. B.) Quantification of TDP-43 inclusions that co-localize with TDP-43 and TIA-1. C.) Incubation in Hanks balanced salt solution for 1 hr induced formation of TDP-43 inclusions (arrows) that co-localized with TIA-1 positive inclusions. Transfected EGFP did not form inclusions under the same conditions. Scale bar: 3 µm.

To test whether inclusions would form in neuronal cells, human BE-M17 neuroblastoma cells were transfected with WT TDP-43, TDP-43_86–414_ or TDP-43_216–414_ constructs N-terminally tagged with EGFP (Fig 1). The TDP-43_86–414_ and TDP-43_216–414_ deletion constructs were designed to correlate with the TDP-43 cleavage fragments of approximately 35 and 25 KD that are present in brain of subjects with TDP-43 pathology [1], [11]. Full length WT TDP-43 predominently localized to the nucleus under basal conditions with only 10% of the cells exhibiting TDP-43 inclusions (Fig. 2A; Supplemental figure S1 shows an example of a cell with some cytoplasmic TDP-43 inclusions). In contrast, about 30% of the cells over-expressing TDP-4386-414formed abundant cytoplasmic inclusions under basal conditions (Fig. 2A). Cells expressing the TDP-43_216–414_ construct also formed inclusions (Fig. 2A), but the number of cells exhibiting fluorescence was low, possibly due to cytotoxicity [11]. The inclusions were predominantly cytoplasmic, but were also present in the nucleus (Fig. 2A).

Many studies suggest that protein aggregation increases under stressful conditions. To investigate TDP-43 aggregation under the stressful conditions, cells were exposed to arsenite, an agent is classically used to induce SGs [16], [17], [18]. Arsenite causes stress through multiple mechanisms [19]. Arsenite directly induces oxidative stress by reacting with oxygen in a reaction similar to the Fenton reaction, and arsenite also uses up glutathione, which causes further oxidative stress [19]. Metabolic responses to arsenite include induction of heat shock proteins, stimulation of NFkB and induction of glucose transporters; the latter change suggests that arsenite might also simulate nutrient deprivation [19]. Each of these changes is known to inhibit protein translation and induce stress granule formation, which makes arsenite an excellent agent for inducing stress granules. Upon exposure to arsenite (0.5 mM, 1 hr) WT TDP-43 remained largely nuclear, but a small amount translocated to the cytoplasm where it formed inclusions (Fig. 2A, right panels, arrows; quantification, Fig. 2B). Inclusion formation was not specific to arsenite treatment because incubation in Hanks balanced saline solution (1 hr, for nutrient deprivation) caused formation of TDP-43 inclusions that also co-localized with the SG marker, TIA-1 (Fig. 2C).

**Figure 3:**
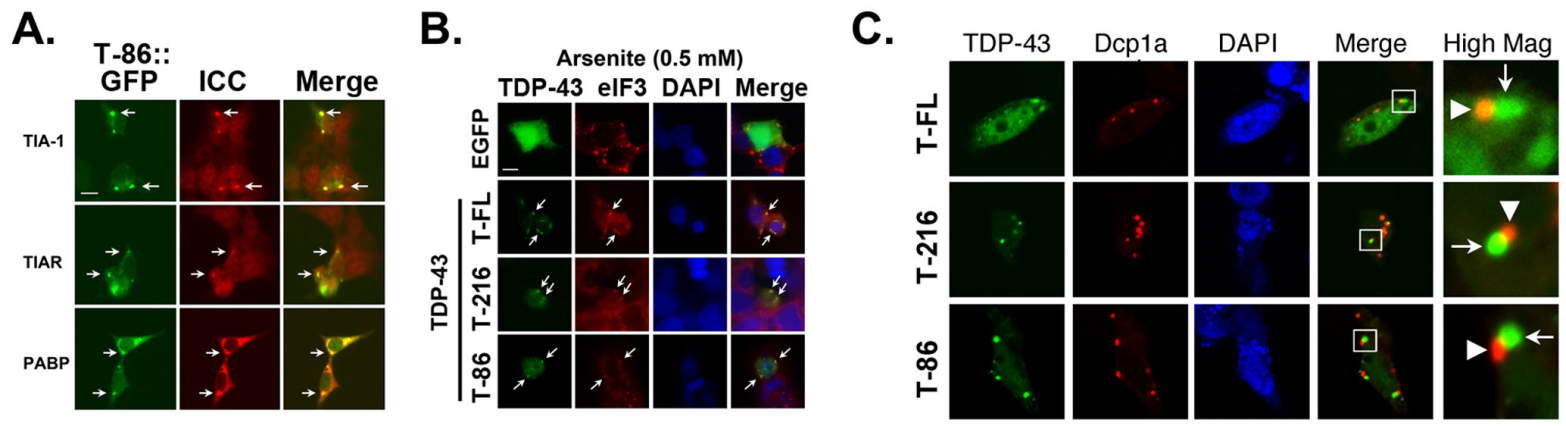
Co-localization of TDP-43 inclusions with SG markers. A.) Human neuroblastoma BE-M17 cells transfected with the TDP-43_86–414_ (T-86::GFP) construct form cytoplasmic inclusions under basal conditions (arrows). The inclusions co-localize with endogenous SG markers including, TIA-1, TIA-R and Poly A binding protein (PABP), identified by immunocytochemistry (ICC). B.) TDP-43 inclusions (WT = T-FL, TDP-43_216–414_ = T-216, TDP-43_86–414_ = T-86, green) occurring after arsenite treatment also co-localize with eIF3 (red). C.) Double-labeling of TDP-43 and Dcp1a (a P-body protein marker). BE-M17 cells were transfected with WT TDP-43::GFP = T-FL, TDP-43_86–414_::GFP = T-86 and TDP-43_216–414_ = T-216::GFP (green) and Dcp1a-mRFP (red). The Dcp1a::mRFP labels P-bodies (triangle), which are adjacent to TDP-43 positive inclusions (arrow). The boxed area is shown in higher magnification in the panels labeled “High Mag”. Scale bar  = 3 µm.

To determine whether the inclusions co-localized with SGs, we co-labeled the cells with antibodies to SG markers, including TIA-1, TIAR, eIF3 and poly-A binding protein (PABP). Double labeling experiments indicated that inclusions composed of WT TDP-43 co-localized with SG markers under arsenite-induced conditions (Fig. 2A, B and 3B, arrows); TDP-43 inclusions also co-localized with SG markers under basal conditions, but the fraction of cells (<10%) exhibiting TDP-43 inclusions under basal conditions. Co-localization with PABP is considered a strong indication of coincident localization of mRNA. Inclusions composed of TDP-43_86–414_ also co-localized with SG markers under both basal and arsenite-induced conditions (Fig. 2A, B and 3A, arrows). Inclusions composed of TDP-43_216–414_ also co-localized with SG markers (Fig. 2A, B), however the fraction of TDP-43_216–414_ inclusions that co-labeled with the SG marker TIA-1 was less than WT-TDP43 or the TDP-43_86–414_ fragment (fig. 2B). Similar results were obtained when experiments were performed using HEK 293 cells (data not shown).

We also explored the relationship between TDP-43 inclusions and P-bodies, which are cytoplasmic protein-RNA complexes that regulate mRNA turnover. We transfected BE-M17 cells with WT, TDP-43_86–414_ or TDP-43_216–414_ fused to EGFP (designated as TDP-43::EGFP, and decapping protein 1a (Dcp1A) fused to RFP (Dcp1a::mRFP), which labels P-bodies. After 24 hrs, the cells were fixed, and examined by confocal microscopy. TDP-43 reactivity for each of the constructs was largely localized in regions adjacent to the Dcp1a/P-body reactivity, which is consistent with prior reports indicating that P-bodies are distinct from SGs, but can align adjacent to SGs and exchange some proteins and mRNA (Fig. 3C, the arrows point to SGs, and triangle points to a P-body) [7], [20]. These data support the hypothesis that TDP-43 can accumulate in cytoplasmic stress granules. However, this does not preclude that TDP-43 can associate with P granules under some conditions because many proteins, such as staufen, FMRP and HuR (but not TIA-1 or eIF3) are present in P-bodies and stress granules depending on the conditions [21], [22].

**Figure 4:**
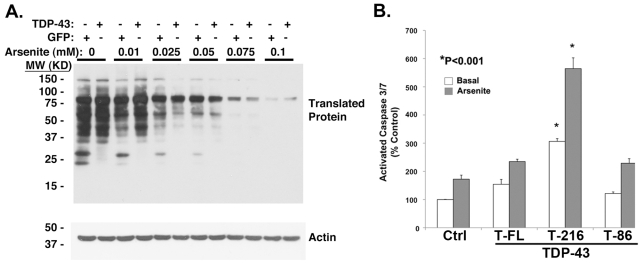
Relationship of TDP-43 to translation and cell death. A.) Representative immunoblot showing the effects of TDP-43 on protein translation. HEK-293 cells were transfected with GFP or TDP-43 (WT, TDP-43_86–414_ or TDP-43_216–414_). After 24 hrs they were treated ± arsenite (0–0.1 mM, 45 min), protein translation was assessed using click-chemistry labeling (15 min pulse). Arsenite treatment strongly inhibited translation of most proteins. TDP-43 expression was not associated with any consistent changes in translation of proteins. B.) Induction of caspase activity upon expression of TDP-43 constructs (WT = T-FL, TDP-43_216–414_ = T-216, TDP-43_86–414_ = T-86); control (Ctrl) refers to transfection with EGFP.

Expression of TDP-43 did not affect total protein translation under basal conditions, where protein translation is robust, or following treatment with arsenite, where protein translation is limited to stress response proteins (Fig. 4A). The experiment was performed by transfecting HEK 293 cells with GFP or TDP-43 (WT). After 24 hrs, the cells were incubated in methionine free medium for 1 hr, then medium containing azido-homoalanine reagent was added to the cells for 15 min. The medium was washed with complete medium, the cells were exposed to arsenite for 45 min, then the lysates were harvested, labeled and immunoblotted. The presence of TDP-43 did not elicit a consistent change in protein translation. A representative protein translation dose-response is shown in Figure 4A; although a small amount of variation in translation was occasionally present between gene constructs at any given concentration of arsenite, there was no consistent effect of the transgenes upon repeat experiments. Formation of the TDP-43_216–414_ inclusions stimulated caspase activity under basal conditions and increased caspase activity after arsenite treatment (Fig. 4B), which is consistent with earlier observations that the 25 KD fragment is particularly cytotoxic [11].

### Endogenously expressed TDP-43 also forms inclusions that co-localize with SGs

**Figure 5:**
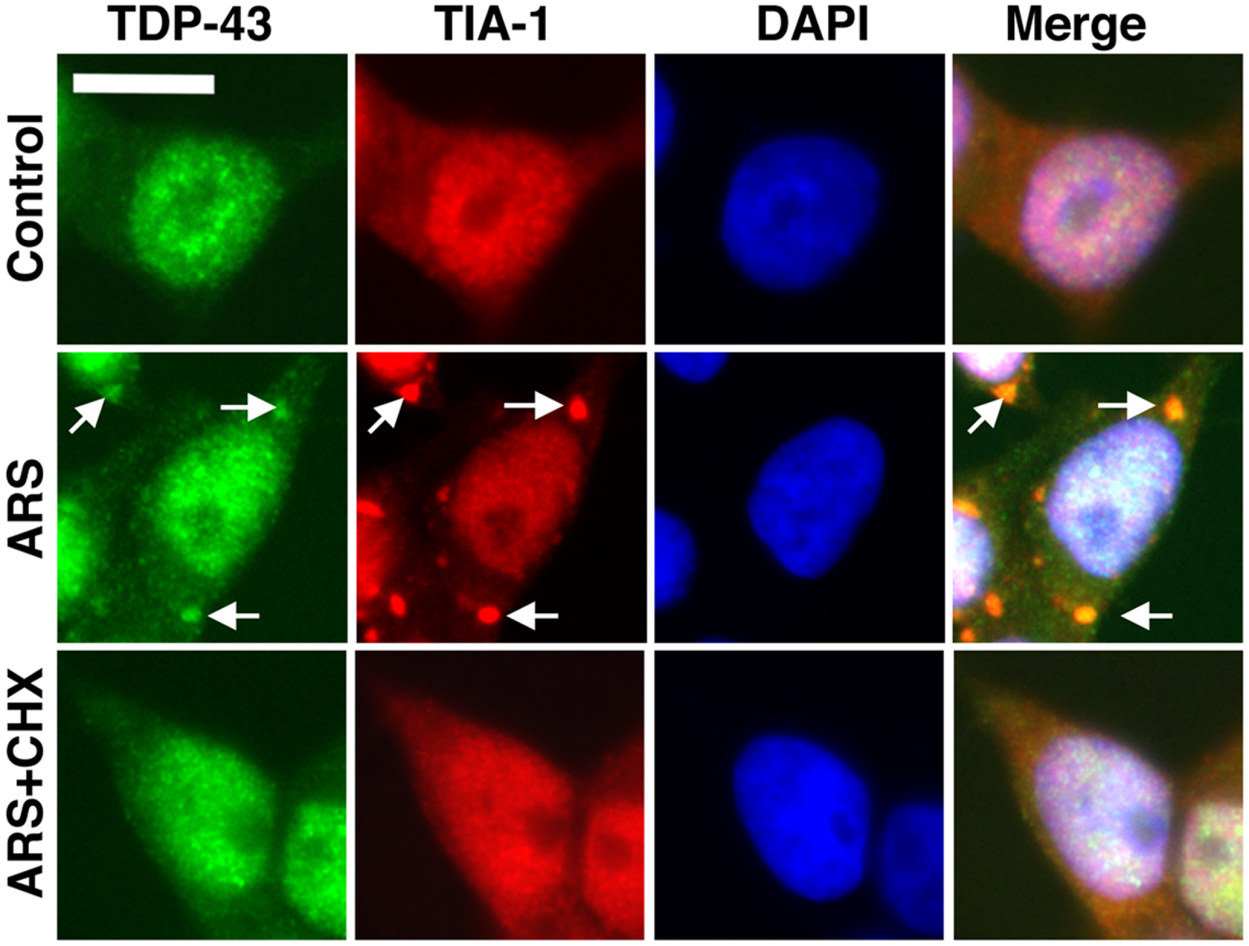
Endogenous TDP-43 forms inclusions after treatment with arsenite (0.5 mM, 1 hr, arrows, BE-M17 cells), and the inclusions are positive for TIA-1 (red). TDP-43 inclusions are suppressed by co-treatment with cycloheximide (50 µg/ml, 1 hr). Inclusions positive for TDP-43 or TIA-1 are not evident under basal (Control) conditions. Scale bar: 10 µm.

Next we examined whether endogenous TDP-43 also formed SG. Human BE-M17 neuroblastoma cells were exposed to arsenite (0.5 mM, 1 hr), the cells were fixed and then immuno-labeled with antibodies to TDP-43 or TIA-1. Most of the endogenous TDP-43 remained nuclear but a small amount translocated to the cytoplasm where co-localization with TIA-1 was observed (Fig. 5, arrows). The amount of cytoplasmic TDP-43 signal was low because labeling only detected endogenous TDP-43, but the TDP-43 that was present in the cytoplasm was largely in inclusions. Concurrent treatment with cycloheximide (50 µg/ml, 1 hr) prevented inclusion formation (Fig. 5). Thus, arsenite-treated cells form inclusions that are positive for TDP-43 and TIA-1 under conditions of endogenous expression or over-expression, and the inclusions are reversible with cycloheximide or emetine. Together these data suggest that inclusions composed of TDP-43 are bona fide SGs.

### TDP-43 binds to TIA-1

**Figure 6:**
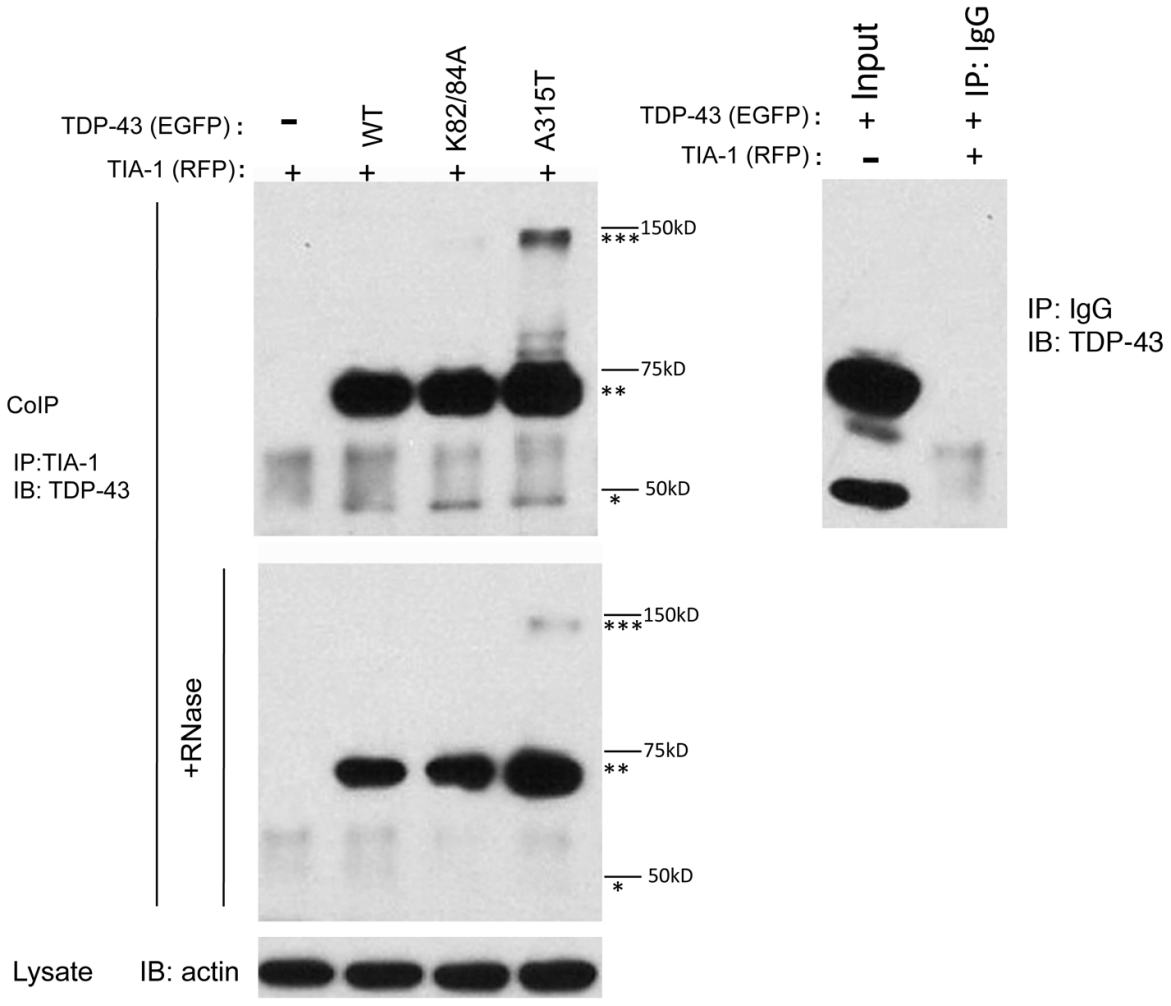
Co-immunoprecipitation of TDP-43 and TIA-1. HEK 293 cells were transfected with TDP-43 (WT, K82/84A or A315T) and TIA-1. The K82/84A TDP-43 construct has had the nuclear localization signal removed (this construct is used again in figure 7B), and the A315T TDP-43 construct contains a mutation associated with familial ALS (this construct is used again in figure 8). The lysates were treated with RNase A (50 µg/ml) for 30 min at 37°C. Immunoprecipitated was performed with antibody to TIA-1, and the immunoblots were probed with antibody to TDP-43. Left **upper panel**: Abundant reactivity corresponding to the TDP-43::EGFP constructs(double star) was evident in lanes co-transfected TIA-1 with TDP-43::EGFP. A weaker band at 43 KD (single star) was also evident, suggesting association between TIA-1 and endogenous TDP-43. Finally, a higher molecular weight band, approximately consistent with the expected size of a TDP-43::EGFP dimer (∼140 KD) was also evident in the lane corresponding to A315T TDP-43 (triple star). **Left lower panel:** Following treatment with RNase A, binding to TDP-43 remained, although it was decreased (double stars). Treatment with RNase A abolished binding between TIA-1 and endogenous TDP-43 (single star), and decreased binding to the upper band (triple star), which presumably corresponds to the TDP-43::EGFP dimer. **Right upper panel**: Negative controls showing the specificity of the immunoprecipitation. Left lane: Lysate (30 µg) of transfected cells used for immunoprecipitation. Right lane: Immunoprecipitation using rabbit IgG instead of anti-TIA-1 antibody. The immunoblot was performed with anti-TDP-43 antibody.

To explore potential mechanisms by which TDP-43 associates with SG, we examined whether TDP-43 binds TIA-1. HEK 293 cells were transfected with TDP-43 and/or TIA-1; co-transfection with TIA-1 induces SGs (data not shown; 293 cells were used because of the higher transfection efficiency). After 48 hrs the cells were lysed and the proteins were immunoprecipitated, using antibody to TIA-1. Cell lysates were treated with or without RNase A before the immunoprecipitation to determine whether any binding interactions were dependent on association with mRNA, and the immunoprecipitated proteins were then immunoblotted with antibody to TDP-43. Co-association of TDP-43 and TIA-1 was readily apparent (fig. 6, upper panel, double stars). A strong band corresponding to the molecular weight of TDP-43::EGFP was evident in lanes co-transfected with TDP-43::EGFP; however, association was not detected in cells transfected with only TIA-1::RFP (fig. 6, upper panel, double stars). Association between TDP-43 and TIA-1 was not abolished by treatment with RNase, suggesting that the two proteins could form a complex that was not dependent on RNA (fig. 6, lower panel, double stars). Binding between endogenous TDP-43 and TIA-1 was also apparent (fig. 6, upper panel, single star), but appeared to be mediated by RNA because RNase treatment eliminated reactivity (fig. 6, lower panel, single star). These data suggest that TDP-43 can bind to TIA-1 at higher concentrations of TDP-43, such as with over-expression, but that binding to TIA-1 is not the predominant mechanism mediating association with SGs under basal levels of TDP-43 expression.

### Formation of cytoplasmic TDP-43 inclusions can be prevented or reversed

**Figure 7:**
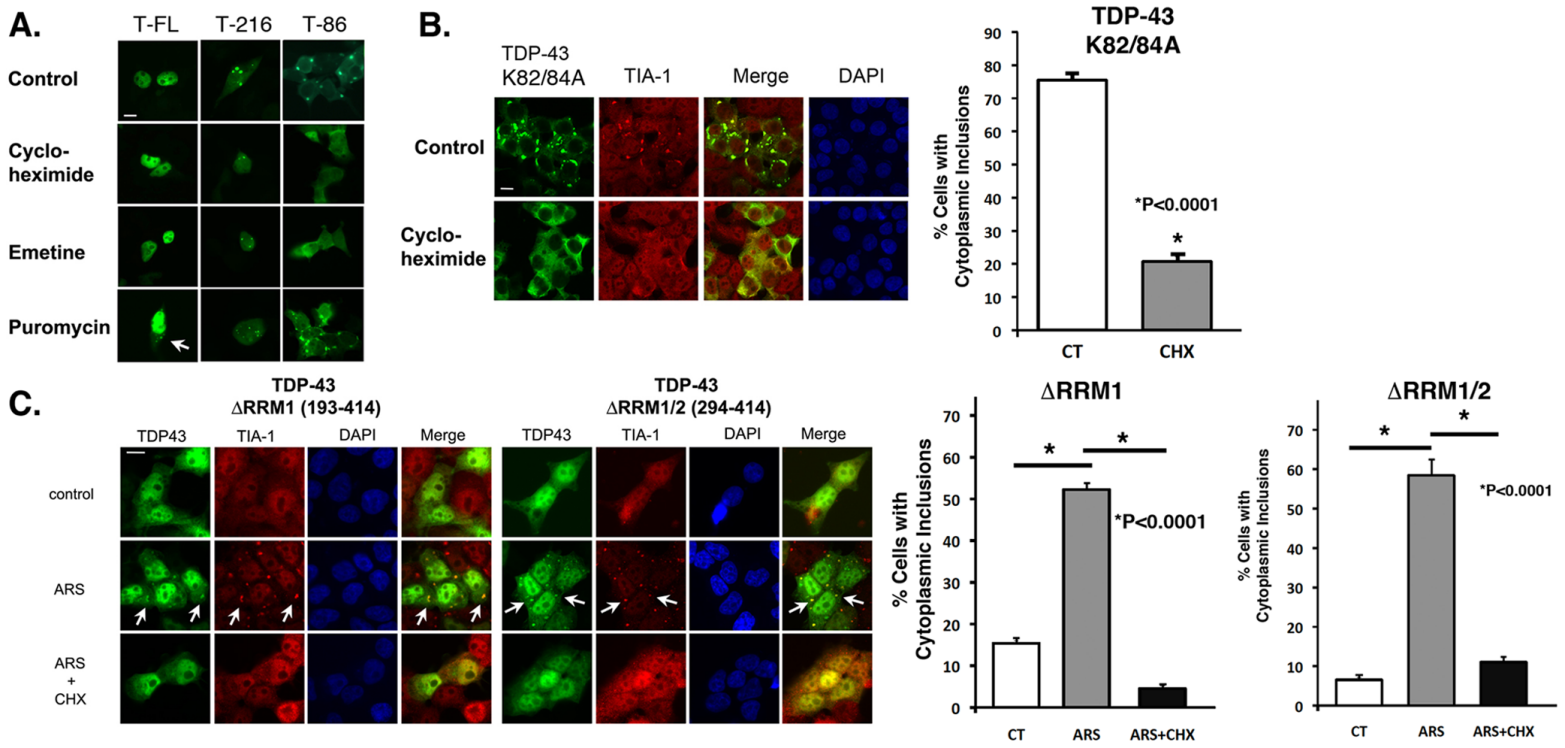
Regulation of TDP-43 inclusion formation by stress granule modulators. A) The translational inhibitors cycloheximide (50 µg/ml, 1 hr) or emetine (20 µg/ml, 1 hr) disperse inclusions formed by WT-TDP-43::EGFP (T-FL) TDP-43_86–414_::EGFP (T-86), while puromycin (20 µg/ml, 3 hrs) increases formation of TDP-43_86–414_::EGFP inclusions formation. Puromycin also induces formation of cytoplasmic inclusions formed by WT and TDP-43_86–414_::EGFP (arrows). However, puromycin does not change the abundance of TDP-43_216–414_::EGFP (T-216) inclusions. B) TDP-43::EGFP lacking the nuclear localization signal (TDP-43 K82/84A) forms abundant cytoplasmic inclusions that co-localize with TIA-1 (anti-TIA-1 antibody, red) after arsenite treatment. The inclusions are dispersed by co-treatment with cycloheximide (50 µg/ml, 1 hr). Quantification is shown in the bar graph to the right. C.) Cells were transfected with the TDP-43 ΔRRM1 and ΔRRM1/2 constructs (N-terminal EGFP tag). After 24 hrs the cells were subjected to two conditions: arsenite (0.5 mM, 1 hr) or arsenite plus cycloheximide (50 µg/ml, 1 hr). The cells were then fixed, and immunocytochemistry was performed TDP-43 inclusions co-localized with TIA-1. Arrows point to representative inclusions showing co-localization of TDP-43 and TIA-1. Cytoplasmic TDP-43 inclusion formation was not apparent under basal conditions, however under conditions of arsenite exposure both constructs formed inclusions that co-localized with TIA-1 and were cycloheximide reversible. Quantification is shown in the bar graphs to the right. Scale bar: 10 µm.

A striking aspect of SG biology is that the inclusion formation is regulated and reversible. SGs form rapidly in response to stress and disperse upon removal of the stress. Formation of SG can be prevented by concurrent treatment with agents that inhibit protein translation but preserve polysomes, such as cycloheximide or emetine [17]. In contrast, SG formation can be stimulated by agents that inhibit protein translation by disrupt polysomes, such as puromycin [17]. Inclusions containing the TDP-4386-414 were responsive to SG modulators. Cycloheximide (20 µg/ml, 2 hr) and emetine (20 µg/ml, 2 hr) dispersed the TDP-43_86–414_ inclusions fully (Fig. 7A); the time course for inclusion dispersion was sufficiently rapid to maintain TDP-43::EGFP expression despite the translational inhibition as demonstrated by the maintenance of GFP fluorescence (Fig. 7A). Conversely, puromycin (20 µg/ml, 3 hrs), which stimulates SG formation [17], increased formation of TDP-43_86–414_ and full-length TDP-43 inclusions (Fig. 7A). Formation of inclusions from TDP-43_216–414_was not very responsive to treatment with cycloheximide, emetine or puromycin (Fig. 7A), which is consistent with the observation that the inclusions composed of TDP-43_216–414_ only partly co-localized with stress granule markers (Fig. 2B).

Because TDP-43 is largely nuclear under basal conditions, we tested whether translocation was required for regulation of inclusion formation. K82/84A TDP-43 lacking a nuclear import signal (Fig. 1) localized to the cytoplasm and formed abundant cytoplasmic inclusions (Fig. 7B), which suggests that elevated cytoplasmic TDP-43 expression stimulates inclusion formation. However, cytoplasmic retention is not sufficient to maintain inclusion formation, because the inclusions can be dispersed by transient treatment with cycloheximide (Fig. 7B).

### RNA binding domains are necessary for inclusion formation

To investigate the role of the RNA binding domains, we deleted one or both RNA binding domains and examined TDP-43 inclusion formation (Fig. 1). Constructs lacking the first RNA binding domain (TDP-43DRRM1) or both binding domains (TDP-43DRRM1/2) did not form inclusions under basal conditions (Fig. 7C). Interestingly, both TDP-43DRRM1 and TDP-43DRRM1/2 did form small cytoplasmic inclusions after arsenite treatment (0.5 mM, 1 hr) (Fig. 7C). The stress-induced inclusions co-localized with TIA-1 and were prevented by concurrent treatment with cycloheximide (20 µg/ml, Fig. 7C). These data suggest that the RNA binding domains can modify inclusion formation under oxidative conditions.

### Knockdown of TDP-43 does not inhibit SG formation

We proceeded to use knockdown experiments to examine whether TDP-43 expression is required for SG formation. In order to characterize knockdown by shRNA constructs, we used HEK293 cells, which have a sufficiently high transfection efficiency to quantify the degree of knockdown by immunoblot (Supplemental Fig. S2A). Next, we knocked down TDP-43 using these shRNA constructs, and identified transfected cells by co-transfection with GFP. After 24 hrs, the cells were treated with arsenite (0.5 mM, 1 hr), labeled with antibody to eIF3, and the number of cells that showed labeling for GFP and eIF3 was counted. Knockdown of TDP-43 did not alter the number of cells positive for stress granules (Supplemental Fig. S2B). These data suggest that TDP-43 does not modulate gross SG formation, but we cannot rule out that modulation might be able to be detected with a more sensitive assay or that TDP-43 modifies the RNA composition of SGs.

### Disease-linked mutations enhance cytoplasmic translocation and SG formation

**Figure 8:**
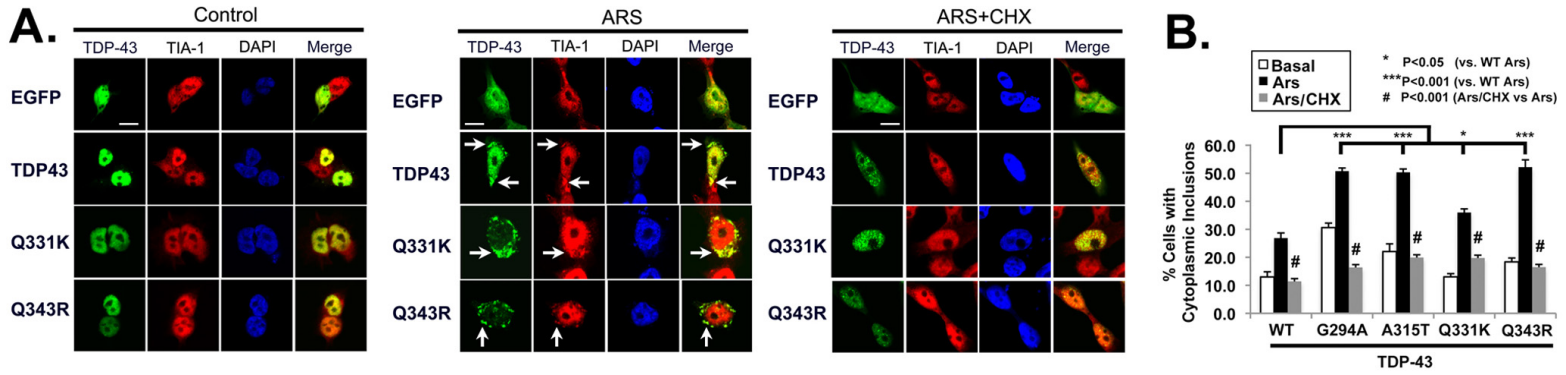
ALS-linked TDP-43 mutations increase aggregation in response to arsenite-induced stress. A) Representative pictures of human neuroblastoma BE-M17 cells transfected with WT, Q331K or Q343R TDP-43::EGFP. The Q331K and Q343R TDP-43::EGFP constructs produce more inclusions (arrows) than WT construct after treatment with arsenite (0.5 mM, 1 hr). However, co-treatment of arsenite and cycloheximide (CHX, 50 µg/ml, 1 hr) prevents formation of inclusions. B) Quantification of cytoplasmic inclusion formation for EGFP and TDP-43-EGFP constructs (WT, G294A, A315T, Q331K and Q343R) under different conditions (basal, arsenite and arsenite + cycloheximide). 15 fields were counted per condition. Scale bar  = 8 µm.

The strong link between TDP-43 and SG biology prompted us to examine whether disease-linked mutations in TDP-43 also enhance formation of inclusions through processes linked to SGs. EGFP-tagged TDP-43 (WT, G294A, A315T, Q331K, Q343R) were transfected into BE-M17 cells, and inclusion formation was examined after treatment with arsenite (0.5 mM, 1 hr) in the presence or absence of cycloheximide (50 µg/ml, 1 hr, Fig. 8). The mutations induced small increases in TDP-43 inclusion formation under basal conditions (Fig. 8B and Supplemental Fig. S3). Arsenite treatment was associated with more inclusion formation for mutant TDP-43 constructs than for WT TDP-43 (Fig. 8A & B and Supplemental Fig. S3). The inclusions that formed in response to arsenite fully co-localized with TIA-1, suggesting that inclusion formed by mutant TDP-43 were also SGs (Fig. 8A and Supplemental Fig. S3). In each case, formation of inclusions composed of mutant TDP-43 constructs was reversed by cylcoheximide (10 µg/ml, 1 hr, Fig. 8A & B). Importantly, each of the mutations also showed a striking decrease in nuclear localization in response to arsenite treatment, suggesting that the mutations increased the degree of nuclear export (Fig. 8A & B). The enhanced stress-induced cytoplasmic localization associated with these mutants might contribute to their strong tendency to form inclusions. These data suggest that enhancement of inclusions with properties resembling SGs is a common feature of TDP-43 mutations associated with ALS.

**Figure 9:**
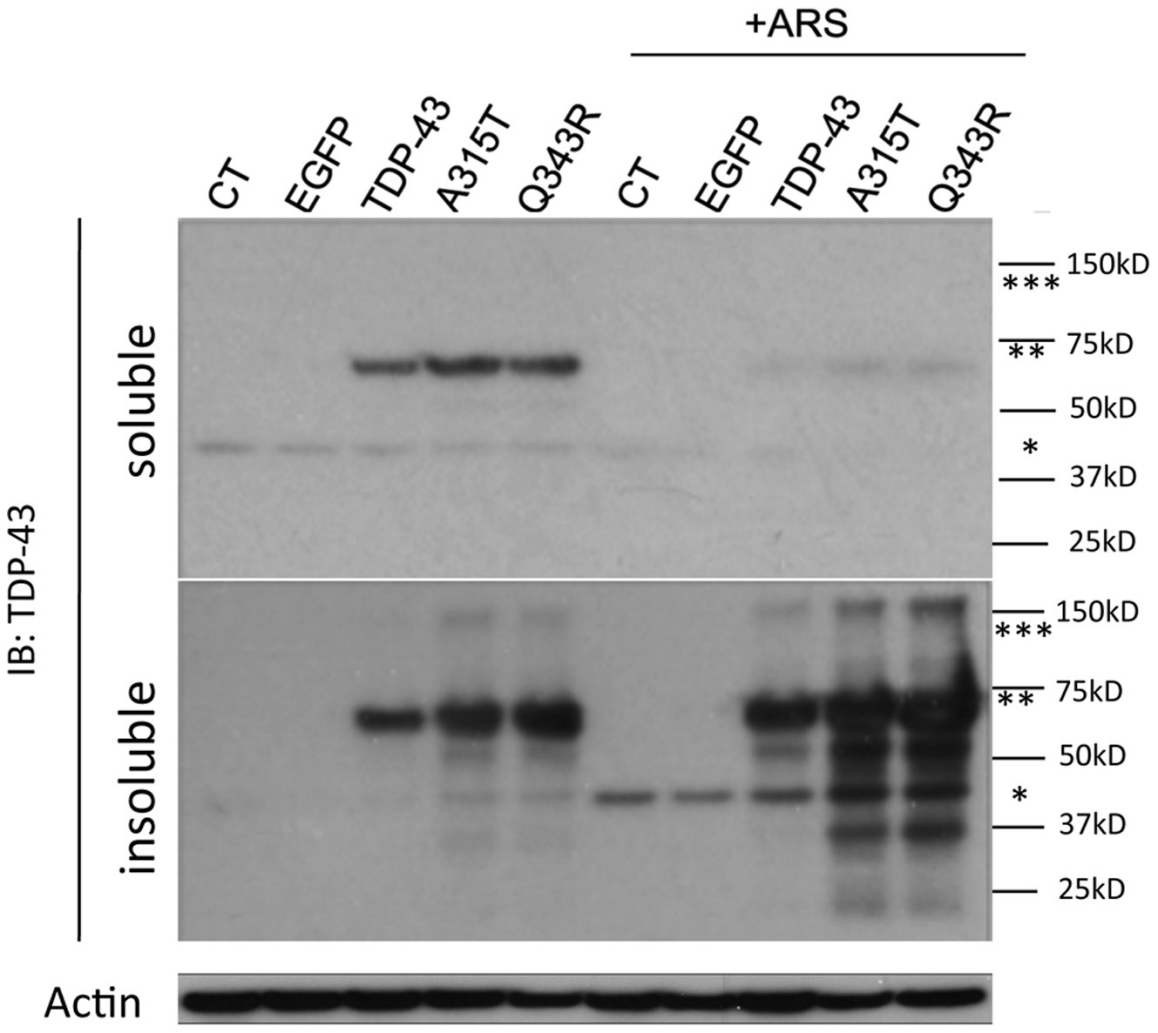
Disease-linked TDP-43 mutations are associated with increased levels of insoluble protein aggregates. HEK 293 cells were transfected with TDP-43 (WT, A315T or Q343R). After 48 hrs, the cells were treated ± arsenite (0.5 mM, 1 hr), separated into soluble and insoluble fractions, and immunoblotted. Upper panel: Soluble fraction. Lower panel: Insoluble fraction. Several different species of TDP-43 could be observed including bands putatively corresponding to endogenous TDP-43 (single star), TDP-43::EGFP (double star) and dimeric TDP-43::EGFP (triple star). Mutations were associated with increased levels of insoluble TDP-43 and increased levels of TDP-43 breakdown products (bands under the TDP-43::EGFP) after arsenite treatment.

Next we investigated the relationship between inclusion formation and solubility because prior studies suggest that TDP-43 forms insoluble inclusions [10], [12]. HEK 293 cells were transfected with EGFP or TDP-43 (WT, A315T or Q343R), grown 48 hrs, incubated ±0.5 mM arsenite, 1 hr, and fractionated into detergent soluble and insoluble fractions. Each fractionation was then immunoblotted. Arsenite treatment increased the amount of insoluble endogenous TDP-43 (Fig. 9, lower panel, CT and EGFP lanes, single star) and transfected TDP-43::EGFP (Fig. 9, lower panel, TDP-43::EGFP, double star). Disease-linked mutant TDP-43 constructs were associated with more increased amounts of total TDP-43 in the insoluble fraction than the WT TDP-43 (Fig. 9, A315T, Q343R, double star, lower panel). Compared to the WT TDP-43, the mutant TDP-43 constructs also showed increased levels of high-molecular weight species, which were present in the insoluble fraction (Fig. 9, lower panels, triple stars). Interestingly, the amount of endogenous TDP-43 present in the insoluble fraction in cells expressing mutant TDP-43 was also increased, suggesting recruitment of endogenous TDP-43 to SGs (Fig. 9, lower panel, single star).

### Disease-linked mutations enhance toxicity

**Figure 10:**
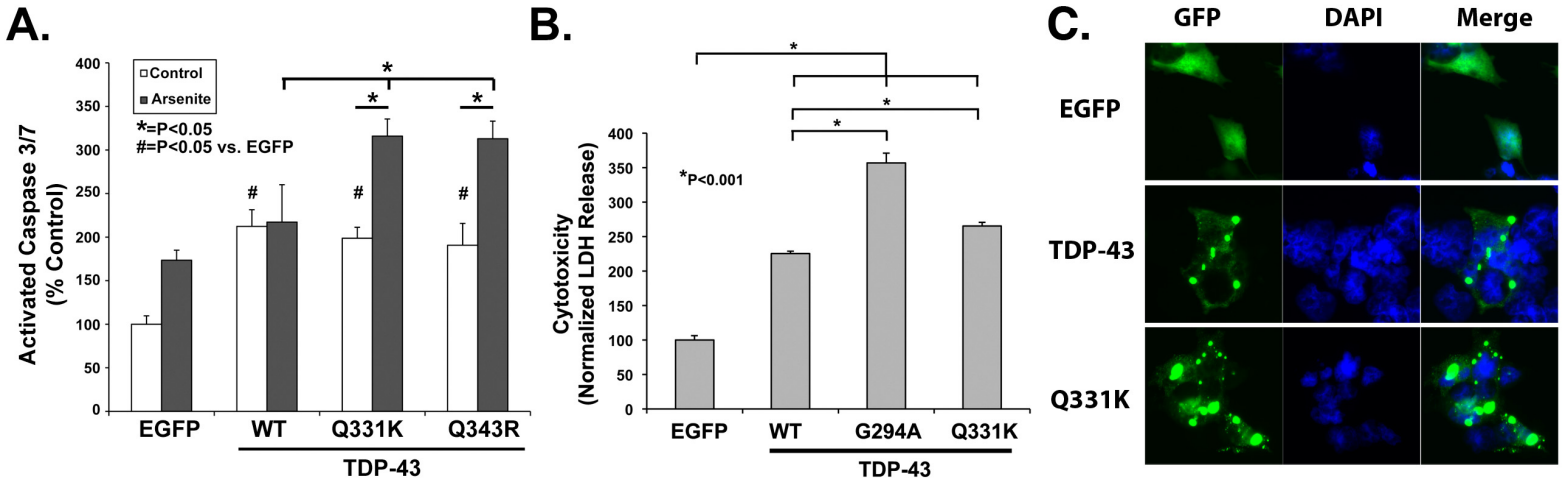
Quantification of toxicity. A) Disease-linked mutations in TDP-43 increase caspase 3/7 activity after arsenite treatment. HEK 293 cells were transfected with EGFP, or TDP43 (WT, Q331K or Q343R). After 48 hrs the cells were treated ±50 µM arsenite, 12 hrs later caspase 3/7 activity was assayed under each condition. Results are shown normalized to EGFP transfected cells under basal conditions. B) BE-M17 cells were transfected with EGFP, or TDP43 (WT, G294A or Q331K). LDH release was measured after 48 hrs and normalized to total LDH and transfection efficiency. Baseline toxicity was set as the EGFP transfected neurons. Transfection with TDP-43 (WT, G294A or Q331K) increased LDH release, with G294A and Q331K TDP-43 causing significantly more release than WT TDP-43 (N = 6 measurements per group). C.) Induction of TDP-43 inclusions by arsenite (50 µM) at 12 hrs.

Previous studies suggest that Q331K TDP-43 expression causes cell death [2]. We hypothesized that the deleterious effects of mutant TDP-43 expression might be linked to SG formation. To investigate the regulation of TDP-43 toxicity, we transfected HEK 293 cells with EGFP or TDP-43 (WT, G294K or Q331K) and measured toxicity using caspase 3/7 activity as a readout. We observed that cells expressing TDP-43 constructs showed elevated caspase 3/7 activity compared to EGFP under basal conditions (Fig. 10A). To investigate the effects of TDP-43 on cell death we examined cells expressing TDP-43 constructs under basal conditions or under conditions in which arsenite induced cell death over 12 hrs. A chronic (12 hrs) stress design was used to facilitate observation of the moderate effects of transgenes, such as TDP-43, on cell death processes. Disease-linked mutations in TDP-43 enhanced toxicity over that with WT TDP-43 after treatment with arsenite (50 µM, 12 hrs, Fig. 10B). Imaging of the cells under these conditions showed formation of TDP-43 positive inclusions (Fig. 10C). Assessment of toxicity depended on the particular assay utilized. For instance, disease-linked mutations in TDP-43 showed greater toxicity under basal conditions than WT TDP-43 with the LDH assay (Fig. 10B). The quantitative differences between the LDH and caspase assays might reflect differential thresholds for the LDH-release or caspase 3/7 activation, or differential signaling for necrosis (LDH assay) versus apoptosis (caspase 3/7 assay). Despite some quantitative differences between different assays, the combined results provide evidence that disease-linked TDP-43 mutations increase cell death processes and SG formation.

### TDP-43 inclusions in brain tissue from ALS and FTLD-U donors co-localize with SG markers

**Figure 11:**
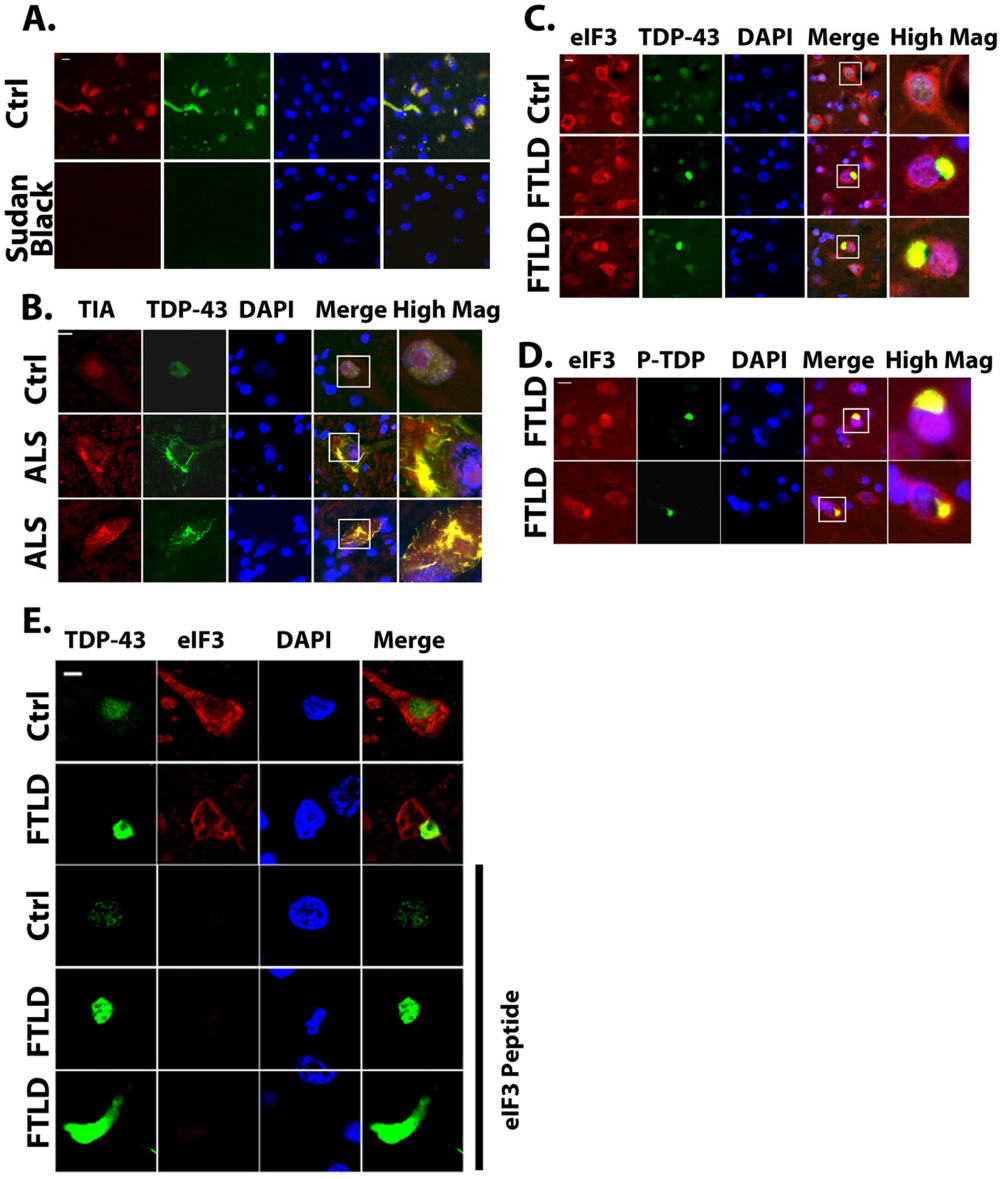
Co-localization of TDP-43 inclusions with SG markers (eIF3 and TIA-1) in ALS and FTLD-U brain. A) Treatment of human cortical brain sections with Sudan black strongly reduced endogenous fluorescence from lipofuscin in the red and green channels (subject 14, Table 1); Blue channel is DAPI staining. B) Co-localization of TIA-1 with TDP-43 in inclusions present in the spinal cord of a patient with ALS (Ctrl  =  subject 5, ALS  =  subject 1, Table 1). The boxed area is shown in higher magnification in the panels labeled “High Mag”. C) TDP-43 inclusions in the frontal cortex of a patient with FTLD-U co-localizes with eIF3 (Ctrl  =  subject 16, FTLD  =  subject 11, Table 1). FTLD-U and ALS sections with TDP-43 inclusions showed co-localization with SG markers. D) Co-localization of phospho-TDP-43 and eIF3 reactivity in FTLD-U brain (subject 10, Table 1). E) Controls for immunocytochemistry. Row 1: TDP-43 present in brain tissue from a patient without neurological disease shows predominantly nuclear localization (subject 14, Table 1). Row 2: TDP-43 positive cytoplasmic inclusion in a patient with FTLD-U showing co-localization with eIF3 (subject 9, Table 1). Rows 3 – 5: Demonstration of immunoabsorption with antigenic peptide, which eliminates staining by eIF3 (Control sample  =  neurological normal, subject 14; FTLD  =  subject 9, Table 1. Scale bars: A, B, C, D = 10 µm, E = 3 µm.

Finally we examined whether TDP-43 pathology present in ALS and FTLD-U cases were associated with SG markers. Immunocytochemistry was performed on cases of ALS and FTLD-U using antibodies to TDP-43 and SG markers, including eIF3 and TIA-1. Sudan black was used to remove endogenous autofluorescence due to lipofuscin (Fig. 11A); this method greatly increased the ability to distinguish between fluorescence related to the antibody signal and fluorescence caused by lipofuscin. Using sudan black to remove autofluorescence, we were able to readily visualize TDP-43 positive inclusions that showed co-labeling with these SG markers in ALS spinal cord tissue and FTLD-U brain (Fig. 11B & C). We also observed co-localization between phospho-TDP-43 inclusions and eIF3 or TIA-1 (Fig. 11D). The specificity of eIF3 staining was tested by immuno-absorption; pre-absorption of TDP-43 antibodies with the antigenic peptide eliminated all reactivity, indicating the specificity of the antibody (Fig. 11E). The absence of reactivity following pre-absorption also demonstrated that labeling of SG markers was not due to the artifact of “bleed-through” from the green channel. No co-labeling was observed with antibody to a different class of RNA-binding protein, the P-body marker anti-Dcp1 (data not shown). In addition, a prior study by Trojanowski and colleagues showed no co-localization of TDP-43 with other RNA binding proteins [23]. Thus inclusions containing TDP-43 in the FTLD-U brain and ALS spinal cord also contain SG proteins, which is consistent with a hypothesis that SG biology is intimately linked to the mechanisms underlying TDP-43 inclusion formation.

## Discussion

Increasing evidence links RNA binding proteins to CNS diseases. Mutations in other putative RNA binding proteins, such as FUS, ataxin-2, SMN and FMRP, are associated with familial ALS, spinocerebellar ataxia, spinal muscular atrophy and fragile X syndrome, respectively [24], [25]. The relationship between SGs and neurodegenerative diseases has been suggested by previous experiments demonstrating that PrP, SMN and huntingtin co-localize with SG proteins in inclusions [26], [27], [28]. In addition, SMN and ataxin-2 both regulate SGs [20], [27]. In the current study, we demonstrate that TDP-43 also co-localizes with SG markers. We demonstrate that mutations in TDP-43 associated with familial disease enhance inclusion formation in response to stress, and also increase cell death. Inclusion formation could be induced by multiple different types of stresses, including arsenite, nutrient deprivation or puromycin. We demonstrate that TDP-43 inclusions are also associated with insoluble protein aggregates. Finally, we show that TDP-43 inclusions in brains of subjects with ALS and FTLD-U co-localize with SG markers. These data suggest that TDP-43 can be added to this group of disease-related proteins whose biology is intimately linked with SGs.

TDP-43 is an intrinsically aggregation prone protein [29]. Structural changes in TDP-43 can increase levels of TDP-43 in the cytoplasm, and these changes appear to stimulate formation of cytoplasmic inclusion. For instance, deletion of the nuclear localization signal in TDP-43 forces expression of TDP-43 to the cytoplasm and causes a concomitant increase in inclusion formation [10]. Cleavage of TDP-43 also produces fragments that exhibit exclusively cytoplasmic localization and a concomitant increase in formation of cytoplasmic inclusions [9], [12]. The A90V mutation increases cytoplasmic localization of TDP-43 and is associated with a corresponding increase in cytoplasmic inclusion formation [30]. Expressing TDP-43 *in vivo* also appears to promote inclusion formation. Over-expressing WT or mutant TDP-43 in mice produces neurodegeneration that is associated with inclusion formation [31], [32].

Our data indicate that TDP-43 can associate with TIA-1, a primary SG protein, however association only occurs upon over-expressing TDP-43. The conditions used to examine the association were conditions in which SG are present because over-expressing TIA-1 induces formation of SGs that include TDP-43. Co-localization of TDP-43 with SG proteins could result from direct binding to SG proteins such as TIA-1, TIAR or G3BP, or via indirect binding mediated by mRNA. Treatment of the TDP-43/TIA-1 complex with RNase eliminated binding of endogenous TDP-43, but did not eliminate binding of over-expressed TDP-43, which suggests that binding can occur, but is concentration dependent. We also noted that TDP-43 constructs showing enhanced inclusion formation also bound TIA-1 in a manner that was resistant to RNase A treatment. Complexes of RNA binding proteins often contain interactions dependent on both protein-protein interactions and protein-RNA interactions. For instance, prior studies of cystic fibrosis transmembrane conductance regulator show that co-association can also occur as part of protein-RNA complexes regulating splicing of genes [5], [33], [34]. Thus, the interaction between TDP-43 and SGs is likely to reflect a mixed mechanism involving both protein-protein interactions, as well as protein-RNA interactions. The type of interaction might depend on the particular species of TDP-43 and on the conditions at the binding site.

We also observed that four different disease-linked mutations in TDP-43 increase cytoplasmic translocation and formation of cytoplasmic inclusions with stress. There was also a modest increase in inclusion formation under basal conditions. Our observation that disease-linked mutations increase cytoplasmic localization concurs with observations by two different groups [30], [35]. The increased inclusion formation was evident using optical methods (immunocytochemistry) as well as through biochemical methods in which we observed that arsenite increased the amount of detergent insoluble TDP-43 and that disease-related mutations increased the tendency of TDP-43 to form detergent insoluble complexes. The analysis in our study adds to these studies by providing comparison of 4 different mutations in one study, while the other two studies each examined a single familial mutant construct [30], [35]. These results suggest that enhanced cytoplasmic translocation might be a general mechanism by which mutations in TDP-43 stimulate inclusion formation. Whether the enhanced cytoplasmic translocation is responsible for the neurotoxicity remains to be determined. Our preliminary studies of cell death, using the LDH and caspase assays, suggest a correlation between cytoplasmic translocation and cell death. Interestingly, Barmada et al observed that cytoplasmic translocaton, but not inclusion formation correlated with toxicity [35]. Proof of this hypothesis requires an experimental paradigm that inhibits cytoplasmic TDP-43 translocation without changing the levels of TDP-43 (our current tools, cycloheximide and emetine modulate translation).

Co-localization of TDP-43 with SG markers is a robust observation that can be observed with multiple independent markers. We observed co-localization using antibodies to eIF3, TIA and PABP. These observations are consistent with those of Colombrita et al, who also observed co-localization of TDP-43 with SGs [15]. The role of TDP-43 in SG biology remains to be determined, because neither we nor Columbrita et al observed any affect of TDP-43 on general RNA translation [15].

The relevance of the link between TDP-43 and SGs is supported by our studies of brain tissue of subjects with ALS or FTLD-U. We observed co-localization under conditions of endogenous expression of TDP-43 and co-localization of TDP-43 in brain tissues from subjects with ALS and FTLD-U. We used antibodies to TIA-1 and eIF3, and validated our work by demonstrating that pre-absorption of eIF3 or TIA-1 eliminated the staining, which proves the specificity. Volkening and colleagues also observed co-localization between TDP-43 and the SG proteins, staufen and TIA-1; they noted that the stress granules were more abundant in ALS tissues [36]. On the other hand, Columbrita et al were able to observe co-localization in cell culture under conditions of over-expression but not in brain tissue [15]. An important difference between our study and that of Colombrita appears to be the methods of detection. We optimized methods to reduce background and maximize the specificity of staining. These methods included use of glycine for antigen retrieval and sudan black for reducing autofluorescence. Tissue not treated with with Sudan black and glycine showed extensive autofluorescence (Fig. 11A). This autofluorescence confounds studies attempting to detect co-localization of antigens by immunofluorescence by creating fluorescent signal that is not due to the antibody reactivity. Neither of these methods were used in the paper by Columbrita et al [15]. The absence of techniques reducing autofluorescence (from lipofuscin) might have contributed to a high level of background autofluorescence and low specific antibody fluorescence signal [15]. Both problems would interfere with the ability to detect co-localization of TDP-43 with SG markers [15]. In contrast, the co-localization that we observed was robust and the pattern of staining was consistent in appearance with that shown in other studies of TDP-43 aggregation using colorimetric methods [1], [37], [38]. Use of colorimetric methods obviate problems associated with autofluorescence, but provide weaker assessment of co-localization [1], [37], [38]. As independent studies investigate the relationship between TDP-43 inclusions and SG protein in brain tissue, the results will become clearer. At the present time, the data presented in this manuscript strongly suggest that SG biology plays an important role in the pathophysiology of TDP-43 in ALS and FTLD-U.

The suggested relationship between protein aggregation, TDP-43 and SG biology is tempered by a studies in Drosophila in which over-expression of TDP-43 leads to neurodegeneration without any apparent TDP-43 aggregation [39]. In addition, recent studies in transgenic mice showed neurodegeneration with only a small amount of cytoplasmic phospho-TDP-43 reactivity [32], [40], [41]. Neither of the TDP-43 transgenic models have shown increased degeneration associated with use of TDP-43 constructs containing mutations associated with familial ALS. These results might indicate that degeneration associated with TDP-43 is unrelated to the cytosolic inclusion formation, however the dose-dependent TDP-43 neurodegeneration observed might also reflect degeneration associated with over-expression of TDP-43, perhaps related to the RNA directed biological function of the protein. Thus, whether the apparent limited aggregation associated with degeneration in these transgenic models reflects biology relevant to the human disease remains to be determined. In this context, the results from our experiments are notable for their ability to highlight physiological and biochemical effects associated with disease-linked mutations in TDP-43. Future study designs in transgenic animals will need to discriminate between effects associated with over-expression of TDP-43 and effects associated with mutations linked to familial disease.

The importance of TDP-43 for SGs does not preclude a role in other biological processes. TDP-43 has been shown to regulate CFTR exon skipping and RNA processing [4], [5], [33]. TDP-43 also appears to play a role in dendrites where it shows changes in cellular localization that correlate with neuronal activity [21]. Wang and colleagues observed that neuronal activity appeared to stimulate association of TDP-43 with P-bodies, which are structures associated with RNA processing [21]. We observed that TDP-43 largely co-localized SGs, but a small amount of reactivity did appear to co-localize with P-bodies, which raises the possibility that TDP-43 might contribute to multiple different RNA-linked functions.

The observation that TDP-43 binds TIA-1, co-localizes with SGs and can be modulated by factors that regulate SG biology suggests intriguing questions related to mechanisms of neurodegeneration. Protein aggregation is classically considered to occur through random association of like proteins [42]. However, inclusions present in disease commonly contain many different types of proteins. Some of the associated proteins are heat shock proteins and chaperones that might reflect the cellular response to limit protein aggregation [43]. SG biology provides a mechanism for bringing together proteins under conditions that are physiologically designed to promote aggregation. Nucleation of SG proteins in response to stress brings together many proteins that have a strong tendency to form insoluble aggregates as an intrinsic aspect of their biology. Some proteins, such as TDP-43 with disease-related mutations or C-terminal cleavage fragments, or proteins with expanded polyglutamine repeat domains, such as ataxin-2, have a particularly strong tendency to aggregate and form protein aggregates that resist dispersion [20]. A recent study by Gitler and colleagues demonstrated that ataxin-2 binds TDP-43 and expanded polyglutamine domains in ataxin-2 promote formation of TDP-43 inclusions [44]. The group also identified cases of ALS that were TDP-43 positive and associated with expanded polyglutamine stretches in ataxin-2 [44]. SG proteins might enhance inclusion formation related to these proteins as a result of their mutual associations.

The biology of SGs also suggests novel approaches for therapeutic intervention. SG nucleation occurs via reversible protein aggregation mechanism that is based on prion domains present in TIA-1, TIAR and G3BP [8]. Our results suggest that the RNA binding domains in TDP-43 also contribute to the regulation of inclusions formation. SG biology represents a compelling example of the biological utility of prion-based protein aggregation. Reversible aggregation has been noted in other systems, such as the interaction of tau with CHIP [45], [46]. We were able to use the reversibility of SG inclusions to suppress or disperse inclusions containing full length TDP-43, and disperse inclusions composed of TDP-43 cleavage fragments. This finding is in agreement with the studies by Colombrita et al [15]. The reversibility of SG biology might also be used to combat FTLD-U or ALS. Achieving therapeutic utility requires as strategy that could selectively inhibit or reverse TDP-43 inclusion formation without impairing SG biology generally, which would be toxic. As the mechanisms underlying the reversible aggregation of SGs become understood, use of SG modulators, rather than full translational inhibitors, might provide sufficient biochemical selectivity much like gamma-secretase modulators can reduce Aβ production without inhibiting the general function of gamma-secretase [47].

## Materials and Methods

### Plasmids

All TDP-43 cDNAs were inserted into a pEGFP-C1 vector at BamH1/XbaI sites. shRNA TDP-43 constructs were in the pLK0.1-puro vector (Sigma). The Dcp1a and TIA-1 cDNAs were inserted into a pRFP vector modified to express monomeric RFP, provided by Paul Anderson (Dana Farber Cancer Institute) and Roger Tsien (UCSD).

### Transfection

DNA was mixed with lipofectamine (Invitrogen) at a ratio of 1 µg/2.5 µl. Most experiments were initiated 24 hrs after transfection, unless otherwise indicated and performed as described previously [48].

### shRNA Knockdown

HEK-293FT cells were grown in Dulbecco's Modified Eagle Medium (DMEM) plus 10% fetal bovine serum and 1% penicillin/streptomycin. For TDP-43, two small hairpin RNAs (shRNAs) were used corresponding to the following TDP-43 domain (amino acids): 927-951 and 1260-1281 (Sigma-Aldrich, Entrez accession number NM_007375). The negative control consisted of a shRNA construct against TDP-43 (corresponding to amino acids 177–197) that was shown to have no effect on transcript or protein levels. Knockdown was performed by mixing 3 µg of shRNA/plasmid with 7.5 µl of Lipofectamine 2000 (Invitrogen) in a final volume of 500 µl of Opti-MEM serum-free medium. HEK-293FT or BE-M17 cells were incubated for 6 hours, and then fetal bovine serum was added to the medium to 10% by volume. Twenty-four hours post-transfection, cells were harvested for Western blot analysis. Cells were lysed on ice in 200 µl of RIPA lysis buffer (10 mM Tris, pH 7.4, 150 mM NaCl, 1 mM EDTA, 1% NP-40, 0.1% SDS, and 0.1% sodium deoxycholate) containing freshly added protease and phosphatase inhibitors, then rotated for 30 min at 4°C. The lysates were then centrifuged at 14,000 rpm for 10 min at 4°C, the supernatant was retained and protein concentration obtained by BCA assay (Pierce).

### Immunocytochemistry

Cells were grown on poly-L-lysine-coated cover slips and were treated as described in the text. Following treatment, the cells were then rinsed with PBS, fixed with 4% paraformaldehyde for 15 min, permeabilized for 15 min with 0.2% Triton-X100/PBS, and blocked for 1 hour in PBS containing 5% donkey serum. Primary antibodies used were: rabbit anti-TDP-43 (1∶1000, Proteintech Group, Inc), rabbit anti-TDP-43, C-terminus (aa 405-415; 1∶1000, Cosmo Bio. Co., #TIP-TD-P09), goat polyclonal TIA-1 (1∶300, Santa Cruz, sc-1751) or eIF3η (1∶300, Santa Cruz, sc-16377). Secondary antibodies were: Dylight 488 conjugated donkey anti-rabbit IgG and Dylight 549 conjugated donkey anti-goat IgG (1∶600, Jackson ImmunoResearch). Primary antibodies were diluted in blocking solution incubated with each coverslip for overnight at 4°C. Cells were then washed three times with PBS and subsequently incubated in secondary antibody for 1 hr, covered from light. Cells were then washed three times with PBS and mounted in Prolong-Gold anti-fade reagent with DAPI (Invitrogen). For quantification, four areas of each cover slip were selected, the number of transfected cells and the number of transfected cells with cytoplasmic aggregates were counted, and the ratio was determined. Each condition was analyzed in triplicate, giving a total of 12 measurements per point.

### Immunohistochemistry of human tissue

Tissue sections were deparaffinized and rehydrated, then treated with 0.1 M glycine for 30 min. The sections were washed 4×5 min in PBS, and microwaved on high in citrate buffer for 10 min. The sections were blocked for 1 hr with 10% donkey serum in 0.2% triton X-100/PBS. Incubation was performed with primary antibodies (described above) overnight in 5% donkey serum/PBS. The sections were then washed 4×5 min, and incubated in secondary anti-goat IgG (1∶600, as above) for 1 hr, washed 4×10 min in PBS/0.02% Tween; the third wash included DAPI (1 µg/ml). Background fluorescence was then quenched by incubating 3 min in 1% sudan black followed by 5 quick dips in PBS and 4×5 min PBS washes with shaking then mounting in Prolong-Gold anti-fade reagent (Invitrogen).

### Microscopy

Microscopy was performed using a Carl Ziss LSM 510 META confocal microscope carrying lasers at 405, 488, 543 and 633 nm. Images were captured using a 40 or 63X lens. LSM proprietary software was used for digital image analysis. Images were combined into figures for the manuscript using Adobe Photoshop software. Images in Supplemental Figure S3 were taken with a Olympus BX-60 (Olympus) equipped with epifluorescence optics, digital camera (AxioCam MRm; Zeiss) and analyzed with Zeiss Axiovision software.

### Caspase assay

HEK 293 cells were used for the assay because of superior transfection characteristics. Cells were grown in 10 cm plates, transfected with TDP-43, trypsinized and plated in 96 well plates using 10,000 cells/well. After 24 hrs, cells were treated with vehicle or arsenite (150 µM or 50 µM). After 6 hrs or 12 hrs the caspase assay was initiated by adding Caspase 3/7 Glo reagent (Promega) and measuring after 45 min.

### LDH assay

293-FT cells were transfected, and after 24 hrs the cells were trypsinized, counted and plated on a 96 well plate at 10,000 cells/well. The following day LDH release was measured using the Cyto Tox 96 NonRadioactive Cytotoxicity Assay Kit (Promega) as per manufacturers directions. Released LDH was normalized to total LDH, and the results were further normalized to transfection efficiency for each plasmid. LDH release from the EGFP transfected cells was set as the baseline (100%).

### Biochemical Fractionation

To examine the effect of arsenite on the solubility profiles of wild type and mutant TDP-43, sequential protein extractions were performed. 293FT Cells were washed twice with cold PBS, lysed in cold RIPA buffer (50 mM Tris-HCl, pH 8, 150 mM NaCl, 1% NP-40, 0.1% SDS, 0.5 mM sodium deoxycholate) with 1x Halt protease inhibitor cocktail (Thermo Scientific) and 1x phosphotase inhibitor cocktail (PhosSTOP, Roche), and sonicated. Protein concentrations were measured by BCA protein assay (Pierce). Lysates were first centrifuged for 30 min at 100,000×g 4°C, and the supernatants were collected as RIPA buffer soluble proteins. To prevent contamination caused by carrying over, the pellets were re-sonicated and re-centrifuged twice at 100,000×g for 30 min at 4°C. RIPA buffer-insoluble pellets were dissolved in urea buffer (7 M urea, 2 M thiourea, 4% CHAPS, 30 mM Tris-HCl, pH 8.5) and sonicated. Soluble and insoluble proteins were analyzed by western blot.

### Immunoblot

Immunoblots were performed using gradient PAGE on a 15-well, 4–20% Tris-Glycine gel (Invitrogen) as described previously [48]. Antibody incubation was at 4°C overnight with rabbit polyclonal TDP-43 antibody (1∶1000, ProteinTech Group) or mouse monoclonal actin antibody (1∶5000, Millipore MAB1501) in TBS-T plus 5% nonfat dry milk.

### Immunoprecipitation

293FT cells were washed twice in cold phosphate-saline buffer (PBS) and harvested by incubating with cold co-IP buffer (20 mM Trish-HCl, pH 7.4, 150 mM NaCl, 1 mM EDTA, 1% Triton X-100, 1x Halt protease inhibitor cocktail (Thermo Scientific) and 1x phosphotase inhibitor (PhosSTOP, Roche)) for 30 min on ice. The protein concentrations were determined by BCA protein assay (Pierce). Equal amounts of lysate were then treated with RNase A (50 µg/ml) for 30 min at 37°C. Lysates were cleared by protein G Dynabeads (Invitrogen) for 1 hour at 4°C; 1 µl of TDP-43 antibody (anti TDP-43 C-terminus 405–414, Cosmo Bio Co.) was added to each cell lysate (300 µg) and the samples were incubated for 3 hours at 4°C on a rotating wheel. 50 µl of protein G Dynabeads was added to the samples, then the samples were incubated for additional 1 hour at 4°C. The Dynabeads were pulled down magnetically and washed three times in co-IP buffer. Eluting buffer (Invitrogen) was added to the protein-bead complexes to elute the binding proteins from the beads. The eluted samples were boiled at 95°C for 5 min in SDS-sample buffer. Proteins were then analyzed by western blot.

### Protein Translation

HEK 293 cells were used (because of the superior transfection efficiency over BE-M17 cells). Pulse labeling was done using the manufacturer's directions for the Click-iT®AHA protein metabolic labeling kit (Invitrogen). Cells were incubated in methionine-free medium for 1 hr, then transferred to fresh methionine-free medium containing the azido-homoalanine reagent and incubated for 15 min, then harvested. For studies using arsenite, cells were incubated in methionine-free medium for 30 min, then arsenite was added and incubation in methionine-free medium continued for another 30 min. Next, the cells were transferred to fresh medium containing the azido-homoalanine reagent plus arsenite and incubated for 15 min, followed by harvesting and measurement of protein levels. The azido-containing proteins were labeled with biotin as per manufacturer's directions. The resulting lysates were separated by PAGE electrophoresis, blocked with 5% BSA and detected with streptavidin-coupled horseradish peroxidase (1∶40,000, 1 hr) followed by enhanced chemiluminescence (Pierce) and exposure to film.

### Tissue samples

All samples used in this study are described in Table 1. Cases of ALS were obtained from the University of Pittsburgh Brain Bank and the Center for ALS Research. Cases of FTLD-U were obtained from the Boston University Alzheimer's Disease Center Brain Bank. Clinical diagnoses were made by board certified neuropathologists according to consensus criteria for each disease. Details on the cases are provided in the table below. Spinal cord tissue was used for the studies of ALS and frontal cortex tissue was used for the studies of FTLD-U. All tissues were paraffin embedded. All human tissues were obtained through a process that included written informed consent by the subjects' next of kin. The acquisition process was evaluated by the Institutional Review Boards (Institutional Review Board of the Boston University Medical Campus and the University of Pittsburgh Institutional Review Board/University of Pittsburgh Committee for Oversight of Research Involving the Dead) and determined to be exempt from review by the full committee.

**Table 1:** Samples used for immunocytochemical studies of TDP-43 in the human central nervous system.

ID	Group	Tissue Source	Gender	Age	PMI	Cause of Death
1	ALS	Spinal Cord	M	67	6	ALS
2	ALS	Spinal Cord	F	79	5	ALS
3	ALS	Spinal Cord	F	63	4	ALS
4	ALS	Spinal Cord	M	67	4	ALS
5	Control	Spinal Cord	F	58	5	Respiratory Failure
6	Control	Spinal Cord	M	48	2	Hepatic Disease
7	Control	Spinal Cord	M	57	2	Pneumonia
8	Control	Spinal Cord	F	53	4	Pulmonary Thrombosis
9	FTLD-U	Frontal Cortex	M	72	5	FTD
10	FTLD-U	Frontal Cortex	M	73	6	FTD
11	FTLD-U	Frontal Cortex	M	73	8	FTD
12	FTLD-U	Frontal Cortex	M	83	20	FTD
13	FTLD-U	Frontal Cortex	M	77	25	FTD
14	Control	Frontal Cortex	F	78	6	Myocardial Infarction
15	Control	Frontal Cortex	F	87	4	Myocardial Infarction
16	Control	Frontal Cortex	M	88	13	Lung Cancer
17	Control	Frontal Cortex	M	94	17	Heart Disease
18	Control	Frontal Cortex	F	87	24	Stroke

## Supporting Information

Figure S1 Picture of the occasional cell expressing WT TDP-43 that has inclusions under basal conditions. The cells were labeled with antibody against TIA-1 to test for co-localization with SGs. Scale bar  = 3 μm.
(3.96 MB TIF)

Figure S2 Knockdown of TDP-43 does not affect SG formation. A) Immunoblot of endogenous TDP-43 in HEK 293 cells following knockdown with shRNA for TDP-43 or negative control; HEK293 was used because of the high transfection efficiency, which facilitates detection of changes by immunoblot. B) Quantification of the number of cells per field with eIF3-positive inclusions, using the experiment described in panel B. 30 fields were counted per condition.
(1.27 MB TIF)

Figure S3 Pictures of human neuroblastoma BE-M17 cells transfected with WT and mutant TDP-43. Cells were transfected with the TDP-43-GFP constructs and then examined under three conditions: basal, arsenite (0.5 mM, 1 hr) or arsenite plus cycloheximide (50 µg/ml, 1 hr). The cells were labeled with antibody against TIA-1 to test for co-localization with SGs. Scale bar  = 3 µm.
(7.23 MB TIF)
