# Correction: Tar DNA Binding Protein-43 (TDP-43) Associates with Stress Granules: Analysis of Cultured Cells and Pathological Brain Tissue

**DOI:** 10.1371/annotation/7d880410-06e3-4fe3-a8f1-e84c89bcf8d0

**Published:** 2011-09-23

**Authors:** Liqun Liu-Yesucevitz, Aylin Bilgutay, Yong-Jie Zhang, Tara Vanderweyde, Allison Citro, Tapan Mehta, Nava Zaarur, Ann McKee, Robert Bowser, Michael Sherman, Leonard Petrucelli, Benjamin Wolozin

The correct 4th author name is: Tara Vanderweyde.

The correct citation is: Liu-Yesucevitz L, Bilgutay A, Zhang Y-J, Vanderweyde T, Citro A, et al. (2010) Tar DNA Binding Protein-43 (TDP-43) Associates with Stress Granules: Analysis of Cultured Cells and Pathological Brain Tissue. PLoS ONE 5(10): e13250. doi:10.1371/journal.pone.0013250 

