# Supplementary figures and images for: Tar DNA Binding Protein-43 (TDP-43) Associates with Stress Granules: Analysis of Cultured Cells and Pathological Brain Tissue
Source: PLoS One. 2010 Oct 11;5(10):e13250. doi: 10.1371/journal.pone.0013250 (PMC2952586; doi:10.1371/journal.pone.0013250)

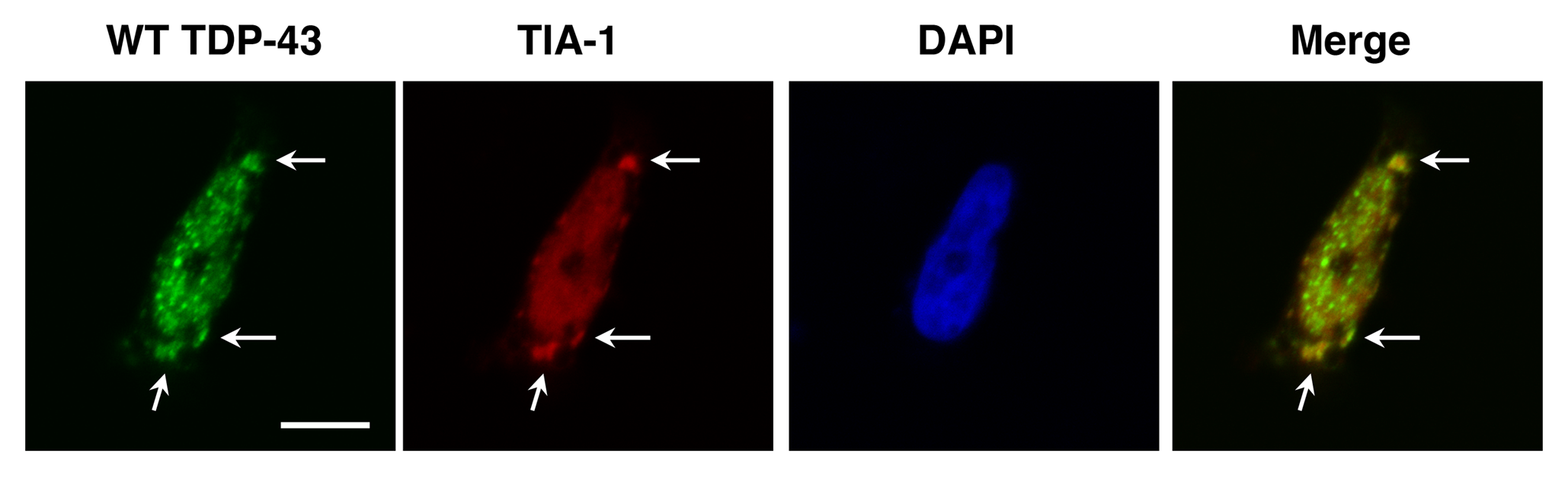

Supplement: Figure S1 — Picture of the occasional cell expressing WT TDP-43 that has inclusions under basal conditions. The cells were labeled with antibody against TIA-1 to test for co-localization with SGs. Scale bar = 3 μm. (3.96 MB TIF) [file pone.0013250.s001.tif]

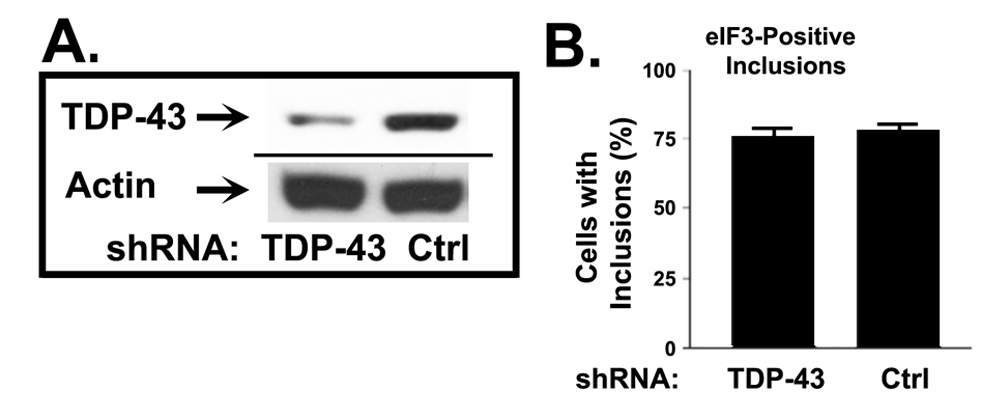

Supplement: Figure S2 — Knockdown of TDP-43 does not affect SG formation. A) Immunoblot of endogenous TDP-43 in HEK 293 cells following knockdown with shRNA for TDP-43 or negative control; HEK293 was used because of the high transfection efficiency, which facilitates detection of changes by immunoblot. B) Quantification of the number of cells per field with eIF3-positive inclusions, using the experiment described in panel B. 30 fields were counted per condition. (1.27 MB TIF) [file pone.0013250.s002.tif]

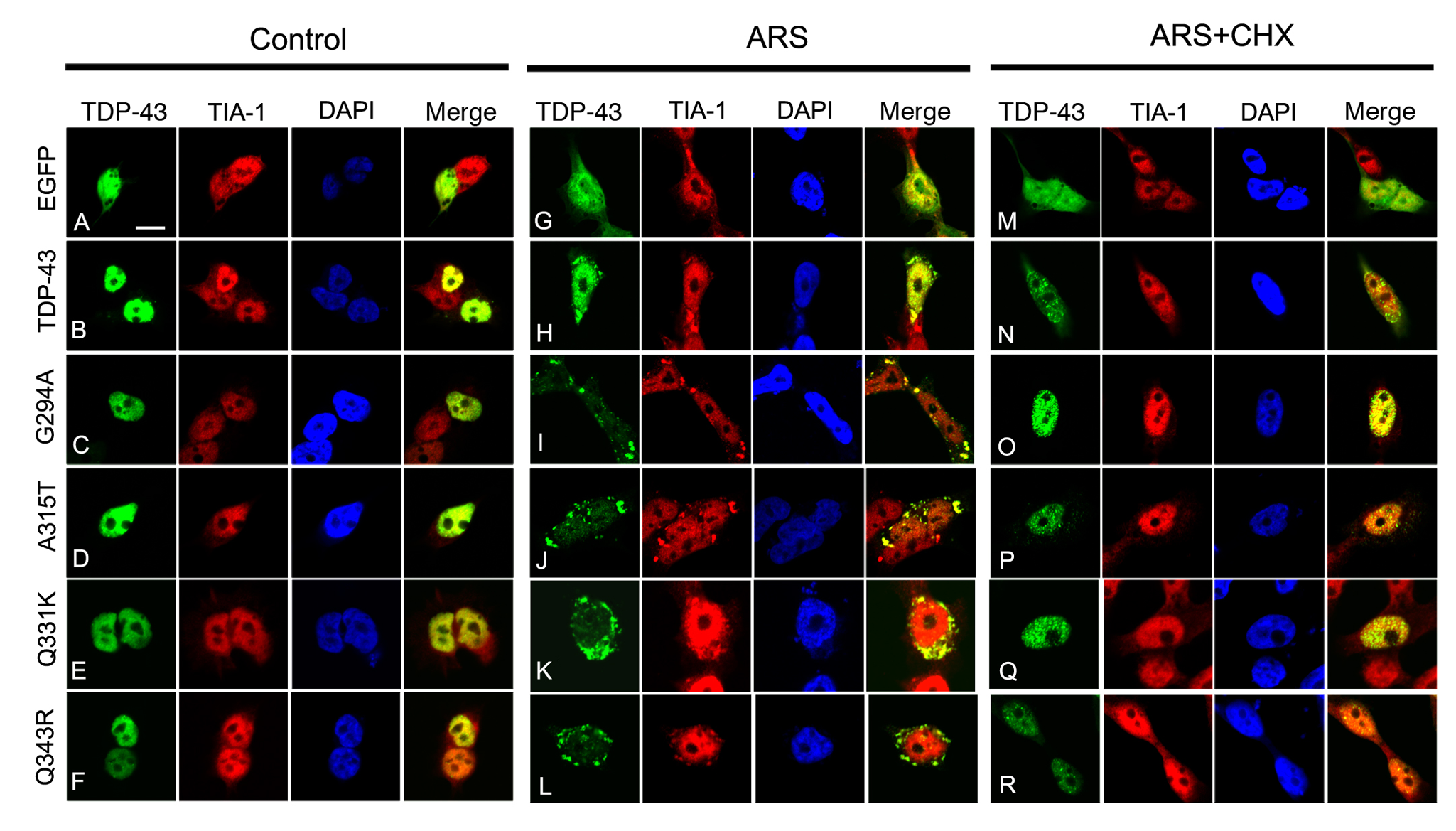

Supplement: Figure S3 — Pictures of human neuroblastoma BE-M17 cells transfected with WT and mutant TDP-43. Cells were transfected with the TDP-43-GFP constructs and then examined under three conditions: basal, arsenite (0.5 mM, 1 hr) or arsenite plus cycloheximide (50 µg/ml, 1 hr). The cells were labeled with antibody against TIA-1 to test for co-localization with SGs. Scale bar = 3 µm. (7.23 MB TIF) [file pone.0013250.s003.tif]
